# Supplementary material for: Distinguishing local isomorphism classes in quasicrystals by high-order harmonic spectroscopy
Source: Nat Commun. 2024 Dec 30;15:10856. doi: 10.1038/s41467-024-55205-x (PMC11685549; doi:10.1038/s41467-024-55205-x)
Supplement: Supplementary file 1 — Supplementary Information [file 41467_2024_55205_MOESM1_ESM.pdf]

Distinguishing Local Isomorphism Classes in Quasicrystals by High-Order Harmonic Spectroscopy: Supplementary Information

Jia-Qi Liu[1] and Xue-Bin Bian[1*]

[1]Wuhan Institute of Physics and Mathematics, Innovation Academy for Precision Measurement Science and Technology, Chinese Academy of Sciences, Wuhan, 430071, China.

*Corresponding author. E-mail: xuebin.bian@wipm.ac.cn

Supplementary Figures

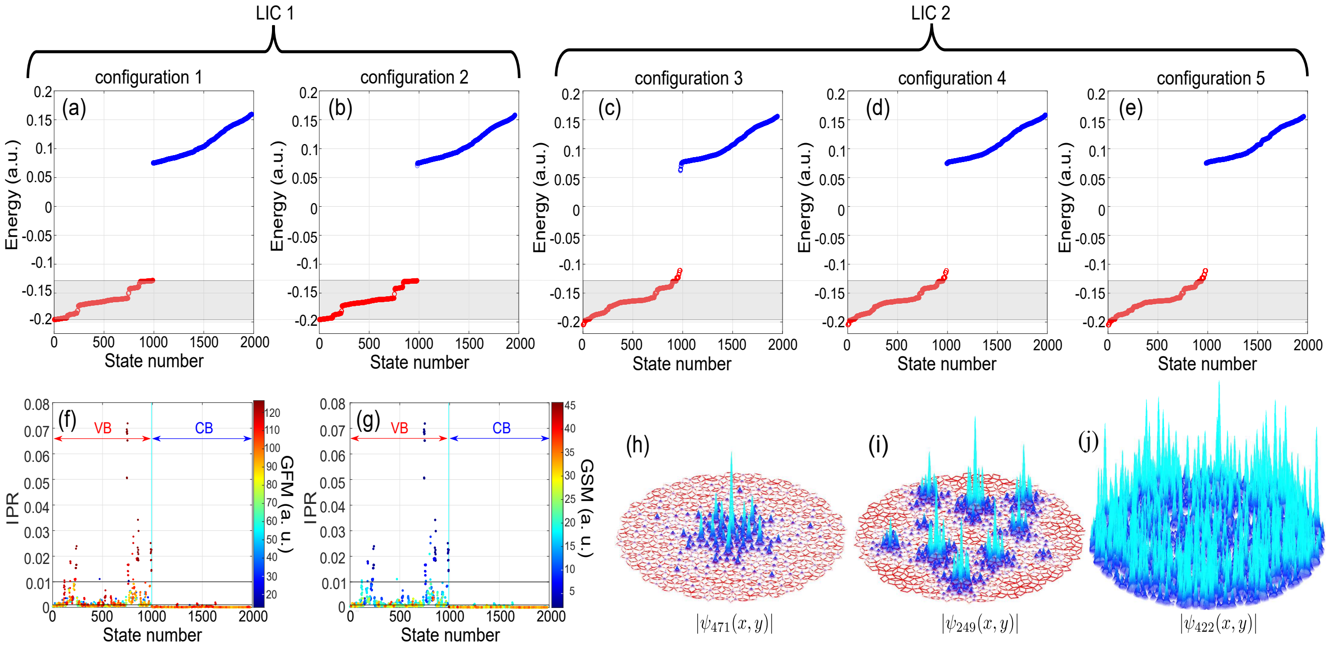

Supplementary Figure 1: **Energy spectra and localization analysis of eigenstates.** (a)∼(e) the energy spectrum of each configuration, divided into two LICs. Taking configuration 1 as an example, (f) and (g) the locality analysis of eigenstate $\psi_i(x, y)$ with IPR, GFM, and GSM. (h) $\psi_{471}(x, y)$, (i) $\psi_{249}(x, y)$, and (j) $\psi_{422}(x, y)$ are the typical local, critical, and extended states of configuration 1, respectively. Source data are provided as a Source Data file.

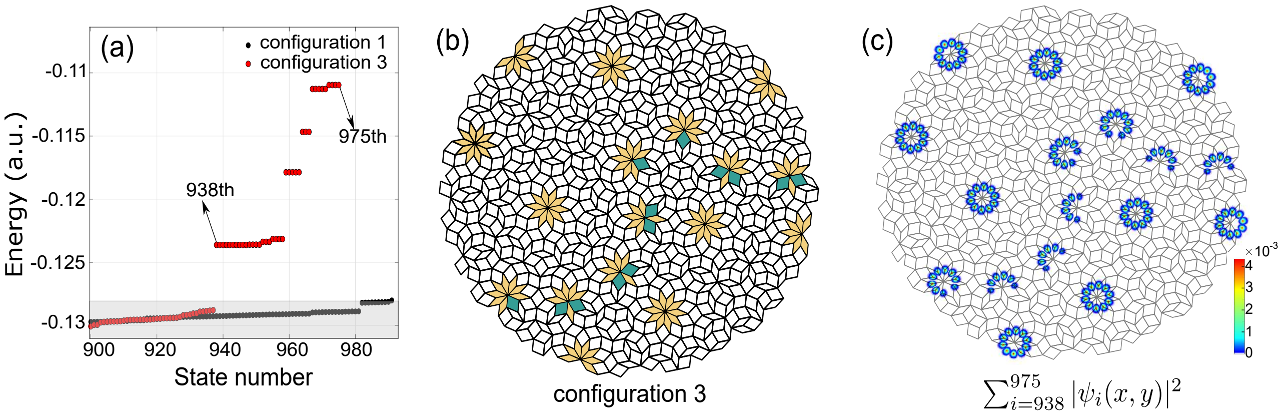

Supplementary Figure 2: **Electron localization due to special VEs.** (a) Energy spectrum comparison between configurations 1 (LIC1) and 3 (LIC2) near the VB top. (b) The distribution of the three type of special VEs in configuration 3. (c) The total electron density of 938th∼975th eigenstates in configuration 3. Source data are provided as a Source Data file.

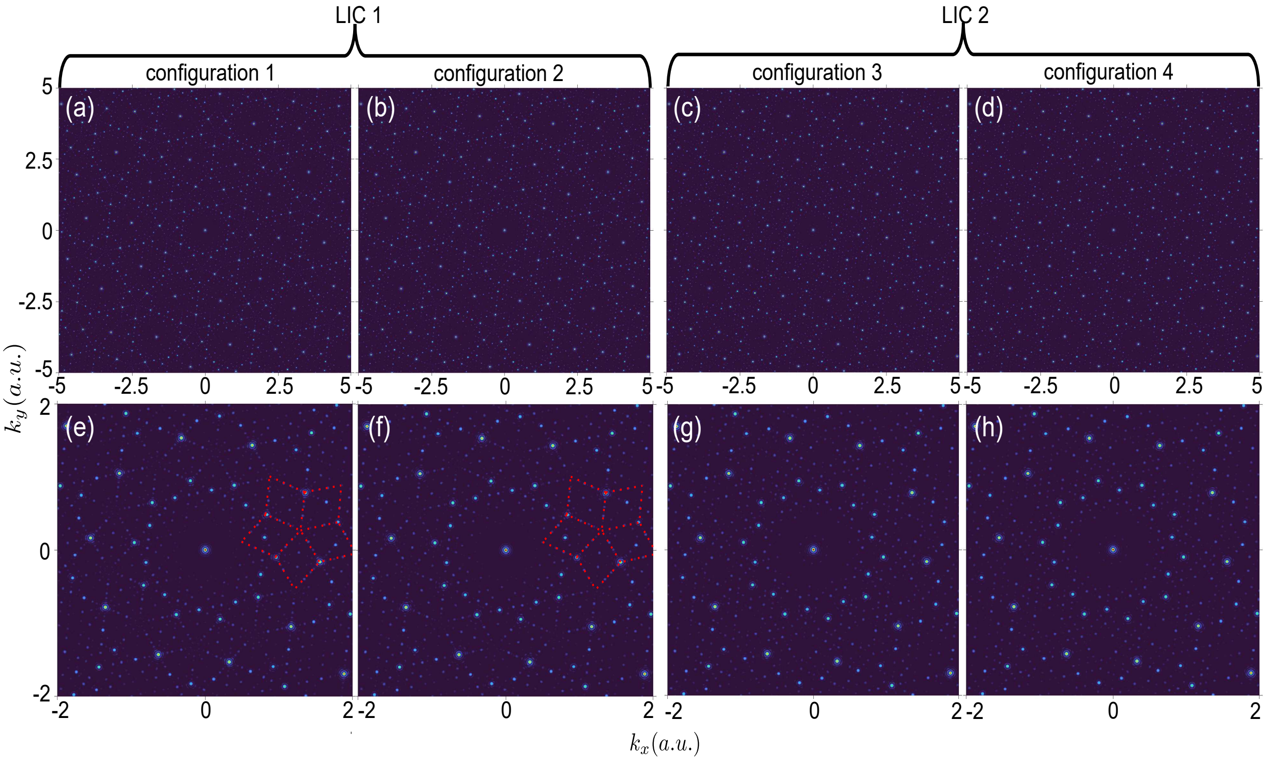

Supplementary Figure 3: **EDSs of 2D generalized Penrose quasicrystals.** (a)$\sim$(d) the EDS of configurations 1$\sim$4, respectively. (e)$\sim$(h) respectively are the partial enlargements of (a)$\sim$(d) for $k_x \in (-2, 2)$ a.u., $k_y \in (-2, 2)$ a.u. Source data are provided as a Source Data file.

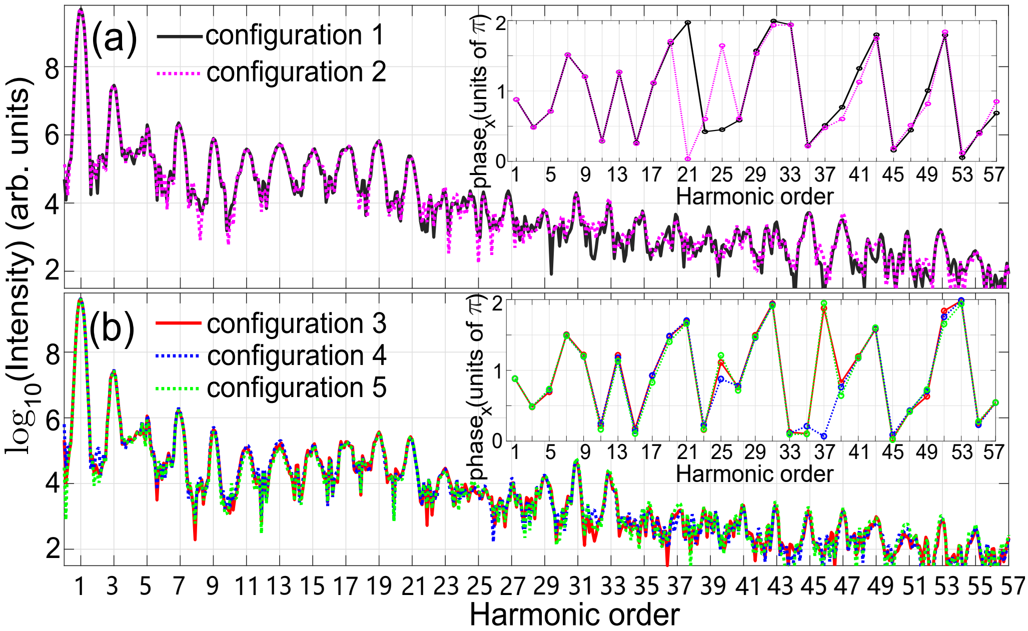

Supplementary Figure 4: **Comparisons of harmonic spectra and phases for different configurations in the same LIC.** (a) The HHGs of configurations 1 and 2 belonging to LIC1. (b) The HHGs of configurations 3, 4, and 5 for LIC2. The laser parameters are consistent with Fig. 2 in the main text. The insets show the odd-order harmonic phases of the corresponding configurations HHGs in the x direction. Source data are provided as a Source Data file.

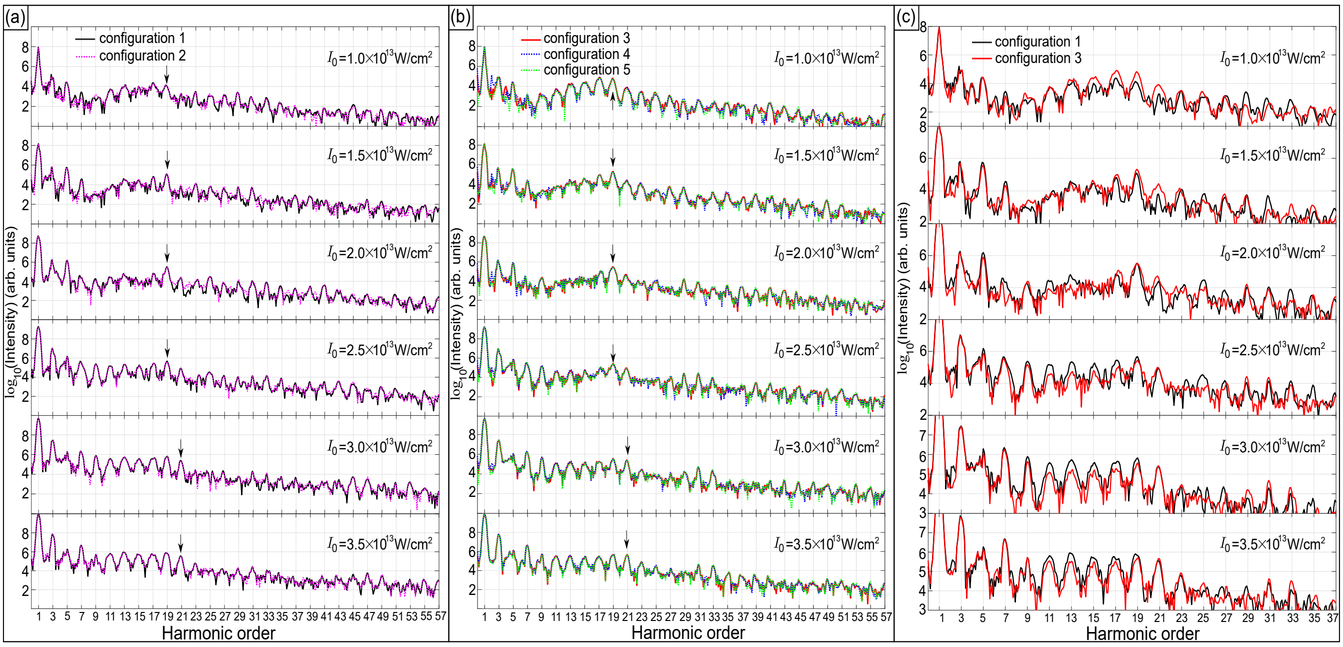

Supplementary Figure 5: **Laser-intensity-dependent HHGs.** (a) HHGs for LIC1 (including configurations 1 and 2) at different laser intensities I_0. (b) HHGs for LIC2 (including configurations 3, 4, and 5) at different I_0. (c) Comparison of HHGs between configuration 1 (LIC1) and configuration 3 (LIC2) at varying I_0. The linear polarization laser is adopted, with the wavelength $\lambda = 2600$ nm. The polarization angle between the polarization direction and the x-axis $\theta = 0$, and the envelope of the $\sin^2$ form, with a total duration of 10 cycles. Source data are provided as a Source Data file.

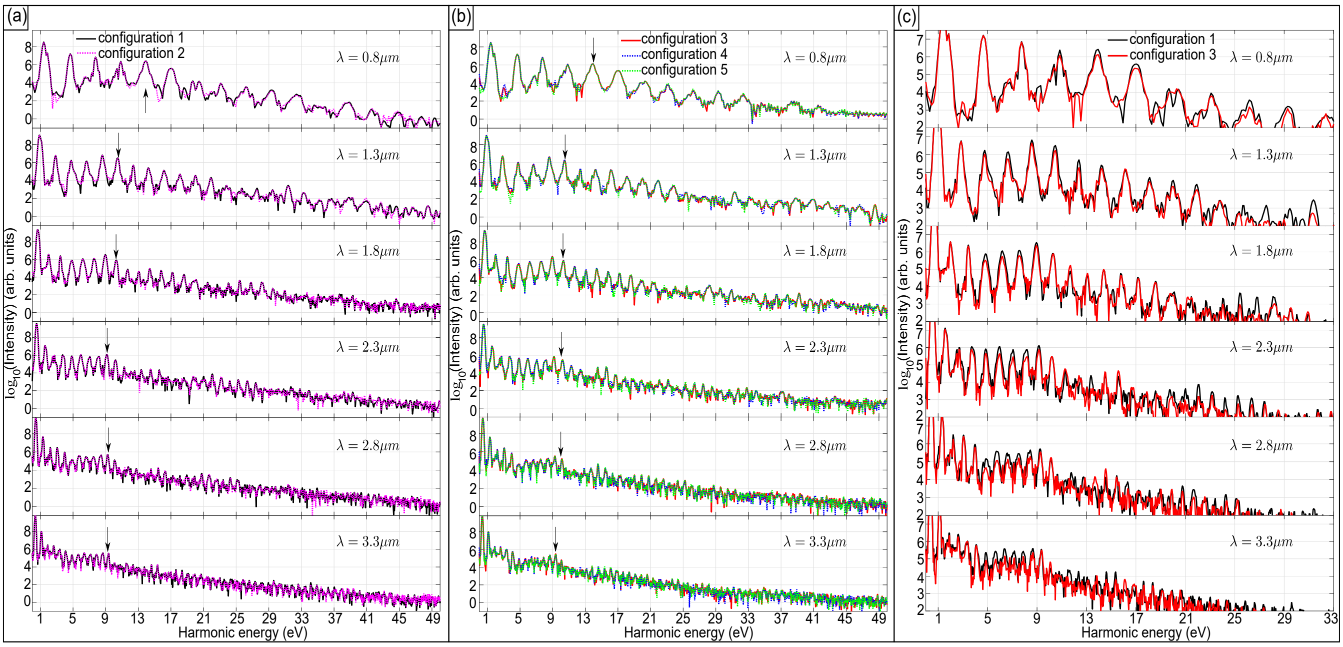

Supplementary Figure 6: **Wavelength-dependent HHGs.** (a) HHGs for LIC1 (including configurations 1 and 2) with different laser wavelengths λ. (b) HHGs for LIC2 (including configurations 3, 4, and 5) with different λ. (c) Comparison of HHGs between configuration 1 (LIC1) and configuration 3 (LIC2) with varying λ. The laser parameters are similar to Supplementary Fig. 5, except for the fixed light intensity $I_0 = 3.0 \times 10^{13}$ W cm^{-2} and the changing laser wavelength. Source data are provided as a Source Data file.

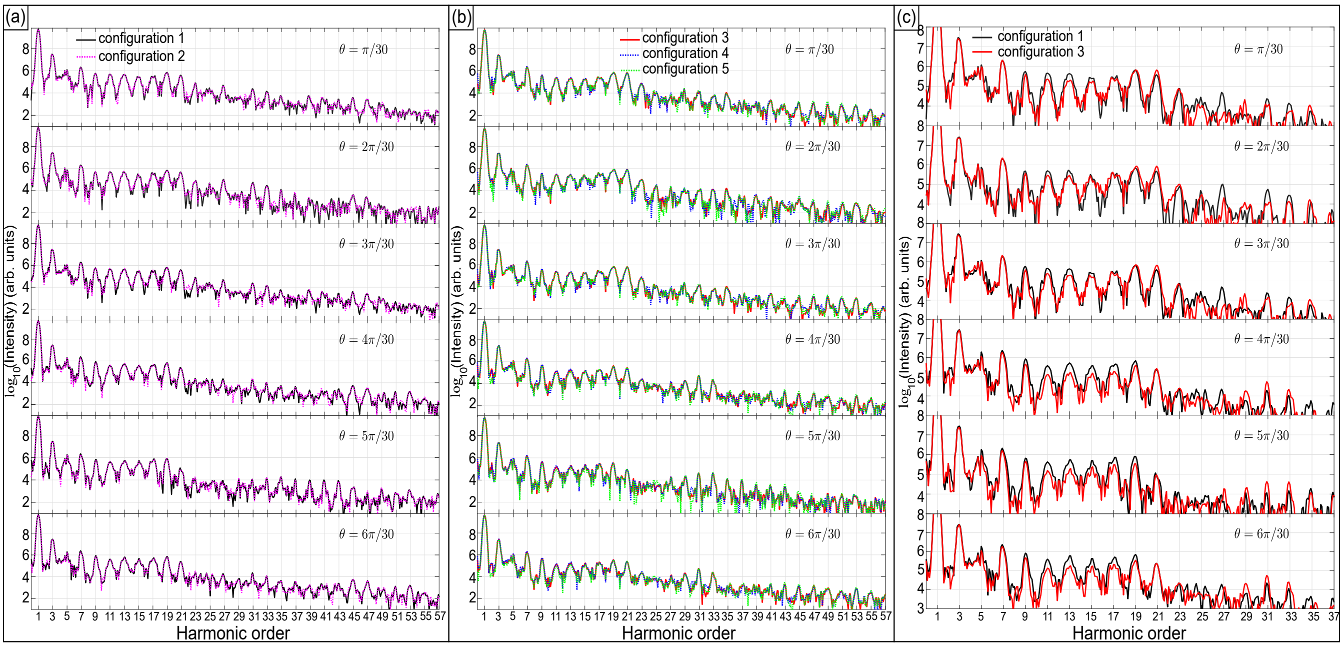

Supplementary Figure 7: **θ-dependent HHGs.** (a) HHGs for LIC1 (including configurations 1 and 2) with different laser polarization angles θ. (b) HHGs for LIC2 (including configurations 3, 4, and 5) with different θ. (c) Comparison of HHGs between configuration 1 (LIC1) and configuration 3 (LIC2) with varying θ. The laser parameters are similar to Supplementary Fig. 5, except for the fixed light intensity $I_0 = 3.0 \times 10^{13}$ W cm^{-2} and the changing θ. Source data are provided as a Source Data file.

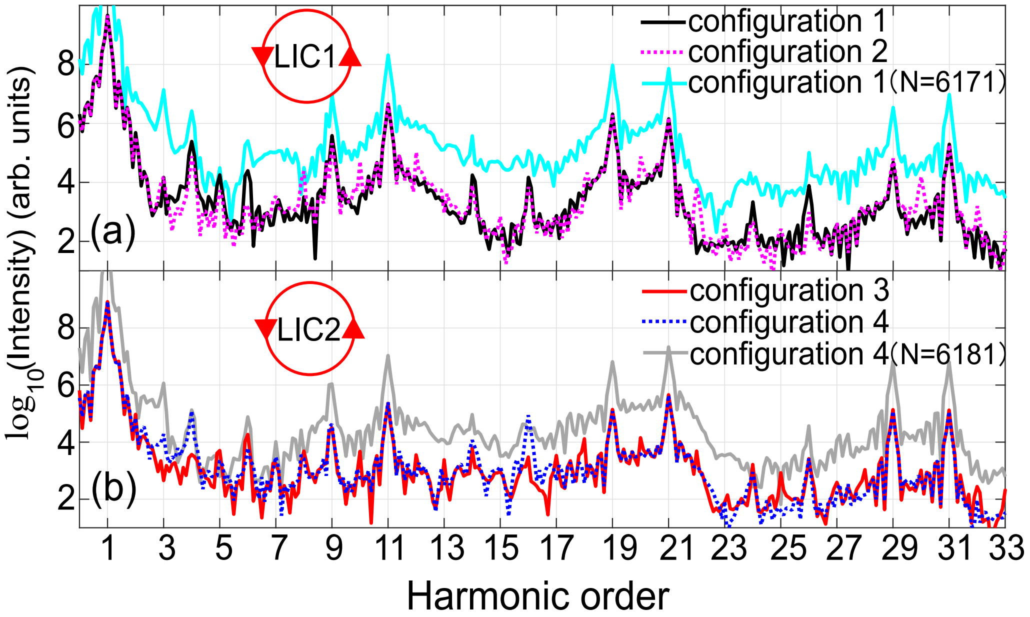

Supplementary Figure 8: **Harmonic radiations driven by a circularly polarized laser.** The HHGs of (a) configurations 1 and 2 belong to LIC1 (with $I_0 = 2.8 \times 10^{13}$ W cm^{-2}), and (b) configurations 3 and 4 for LIC2 (with $I_0 = 2 \times 10^{13}$ W cm^{-2}). A trapezoidal envelope is adopted, including two rising and falling cycles. The harmonic spectrum for configurations 1 and 4, with an increased atomic number ($N_{atom} = 6171$ and $N_{atom} = 6181$), are depicted in cyan and gray, respectively. Source data are provided as a Source Data file.

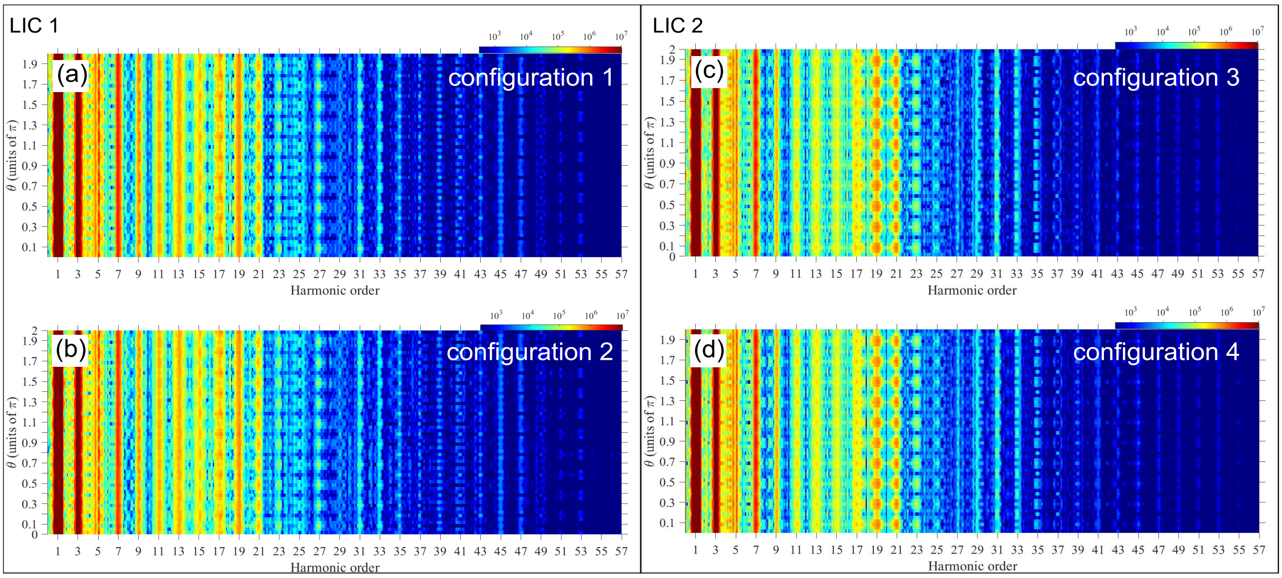

Supplementary Figure 9: **The θ-dependent HHG of configurations 1∼4.** (a) and (b) respectively show the θ-dependent HHG of configurations 1 and 2 (LIC1). (c) and (d) respectively show the θ-dependent HHG of configurations 3 and 4 (LIC2). The laser intensity and wavelength are consistent with Fig. 2 in the main text. The plot in logarithmic scale. Source data are provided as a Source Data file.

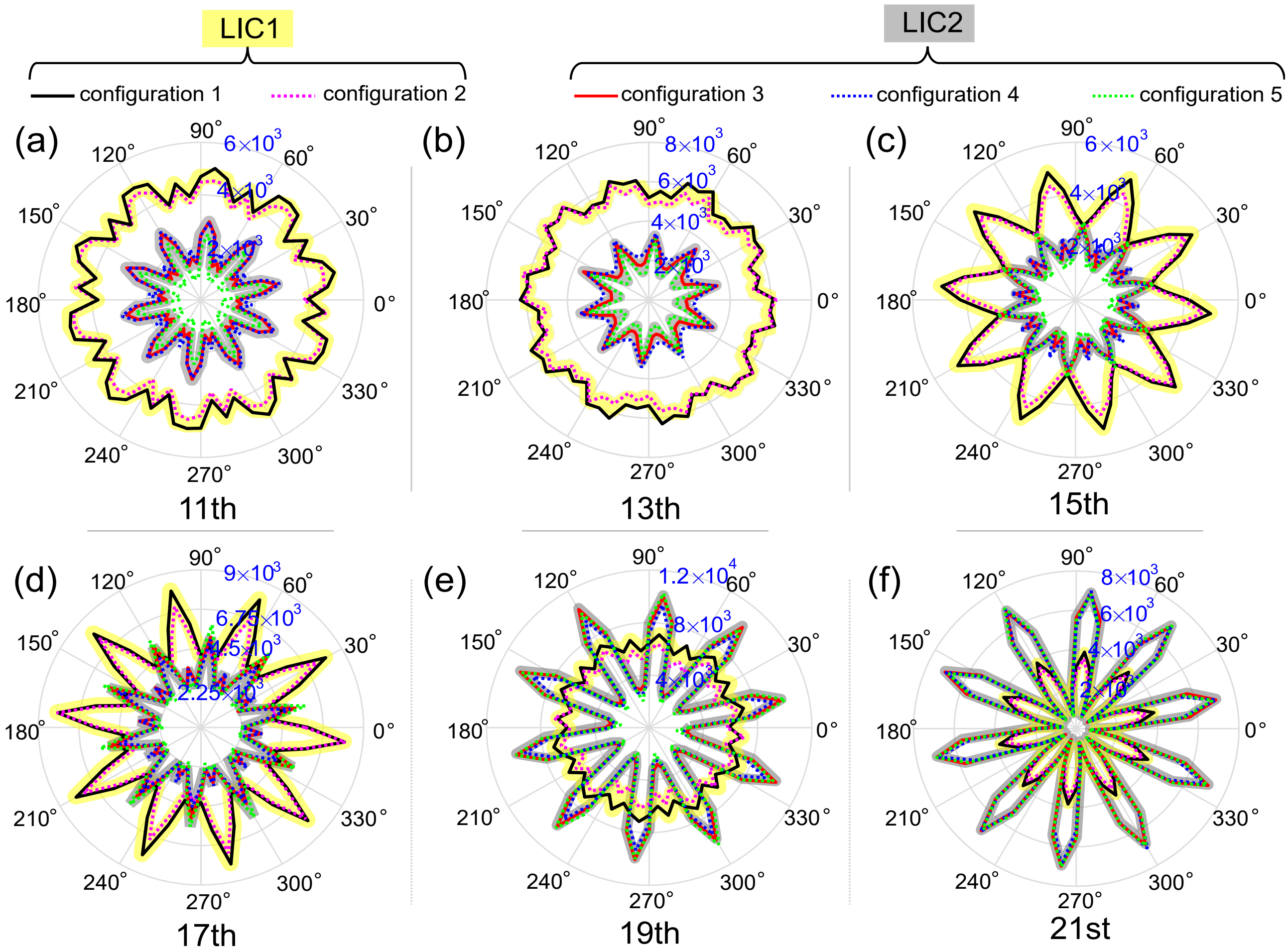

Supplementary Figure 10: **Comparison of orientation-dependent 11th∼21st order harmonic yields for different configurations.** LIC1 (highlighted in yellow): configurations 1, 2; LIC2 (highlighted in gray): configurations 3, 4, 5. (a)∼(f) display the 11th, 13th, 15th, 17th, 19th, and 21st-order harmonic yields for all configurations, respectively. The laser intensity and wavelength are consistent with Fig. 2 in the main text. Source data are provided as a Source Data file.

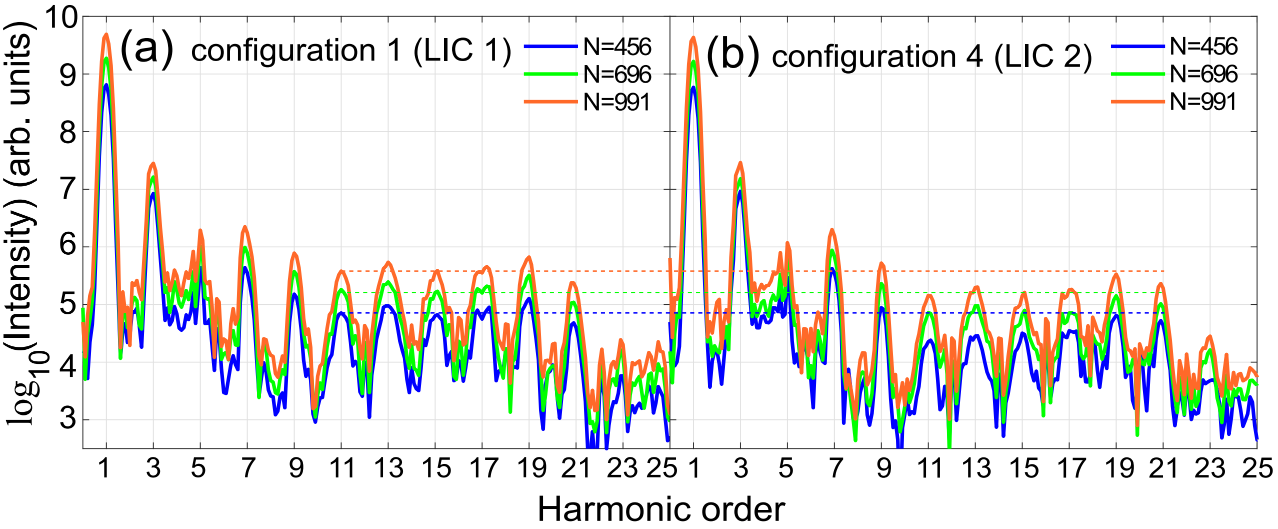

Supplementary Figure 11: **Convergence test of configuration size.** The variation of high harmonic spectra for (a) configuration 1 and (b) configuration 4 with the number of atoms in the system. The laser parameters are consistent with the Fig. 2 in the main text. Dashed lines are used as visual guides to more intuitively illustrate the higher yields of 11th to 19th order harmonic in LIC1 compared to LIC2. Source data are provided as a Source Data file.

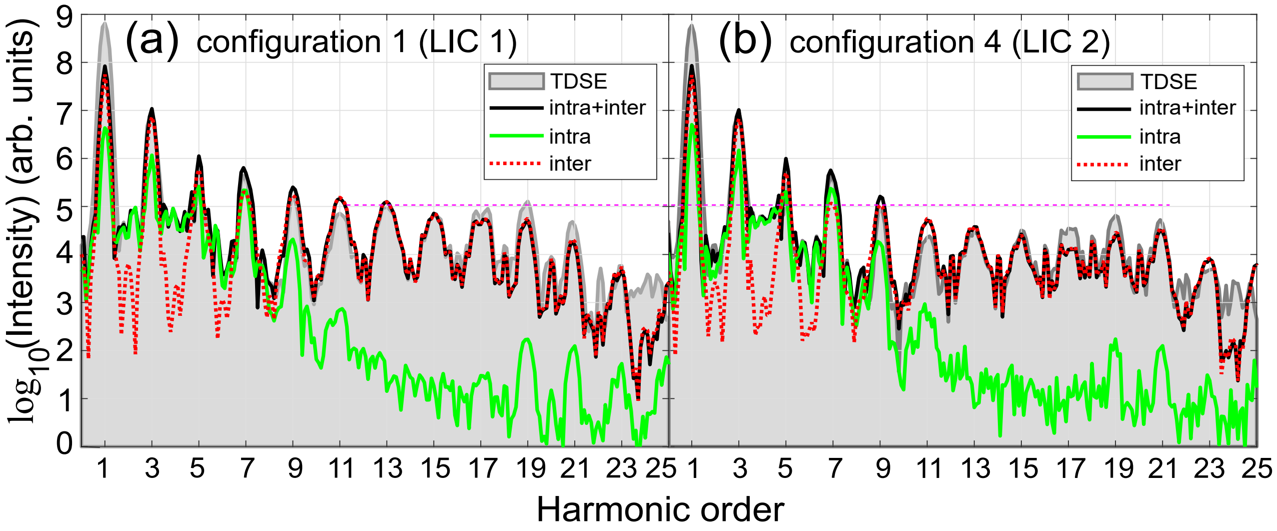

Supplementary Figure 12: **Contribution of inter- and intraband HHGs.** (a) Configuration 1 and (b) configuration 4, with $N = N_e = 456$. Intra, inter, and intra+inter HHGs are shown by the green solid line, red dashed line, and black solid line, respectively. The laser parameters are consistent with Fig. 2 and Supplementary Fig. 11. For comparison, HHG from the standard TDSE calculation is highlighted in gray. Dashed magenta line is also used as visual guides to more intuitively illustrate the higher yields of 11th to 19th order harmonics in LIC1 compared to LIC2. Source data are provided as a Source Data file.

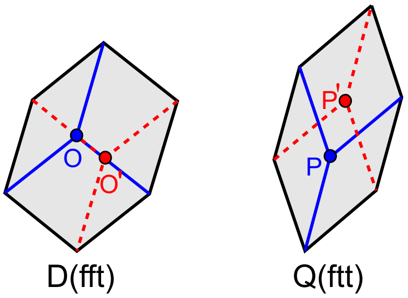

Supplementary Figure 13: **Two possible flips.** For fft (or ftt), VE D's position is shifted from O to O' (or VE Q's position is shifted from P to P').

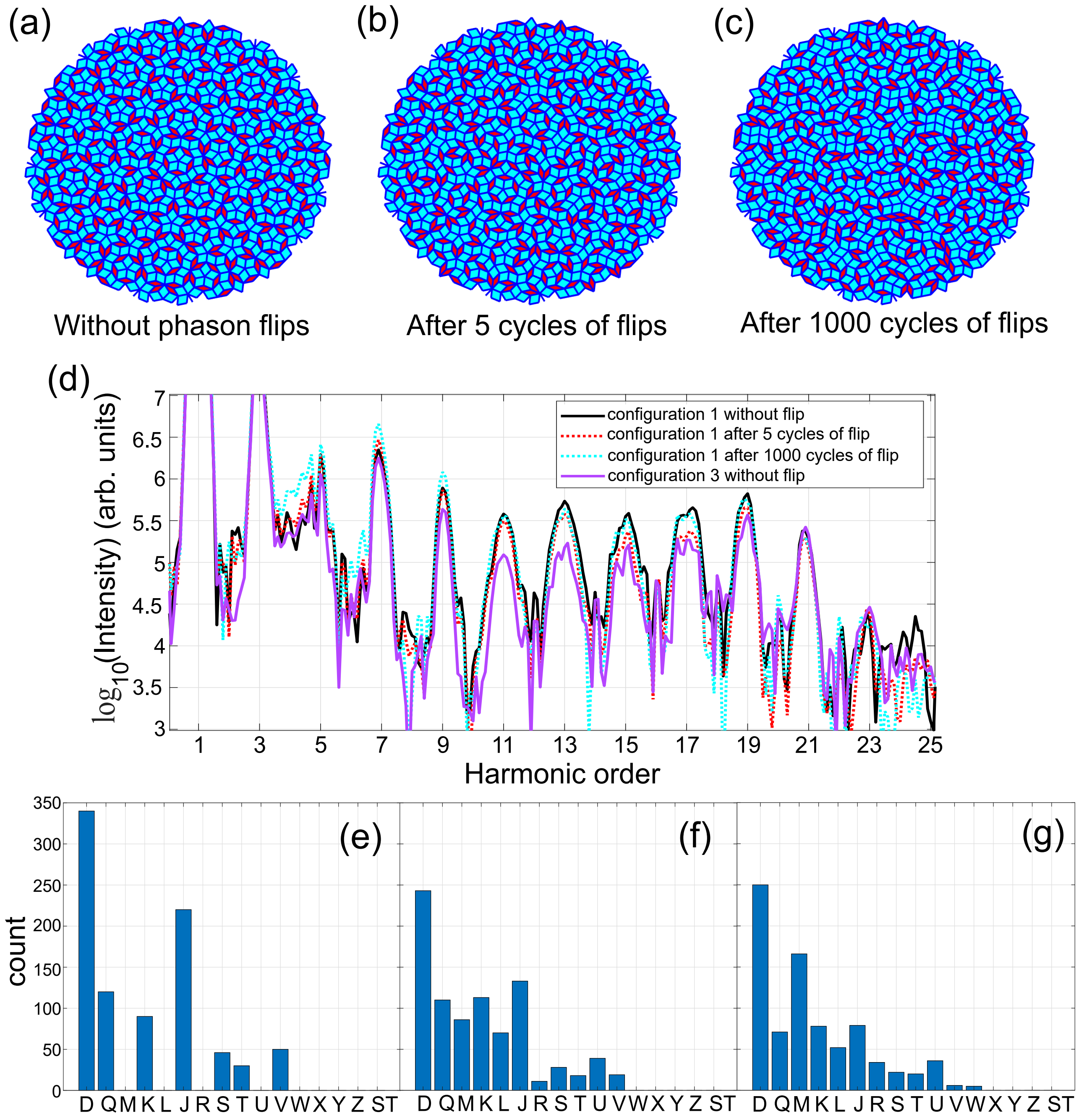

Supplementary Figure 14: **Effect of phasonic disorder on harmonic yields and VE components.** (a)∼(c) The evolution of the tiling of configuration 1 after randomization with an increasing number of phason flip iterations (from left to right). (d) shows the harmonic spectra of configuration 1 after different flip cycles, compared with configuration 3 (LIC2). (e)∼(g) show the statistics of the VE type and count for (a)∼(c), respectively. Source data are provided as a Source Data file.

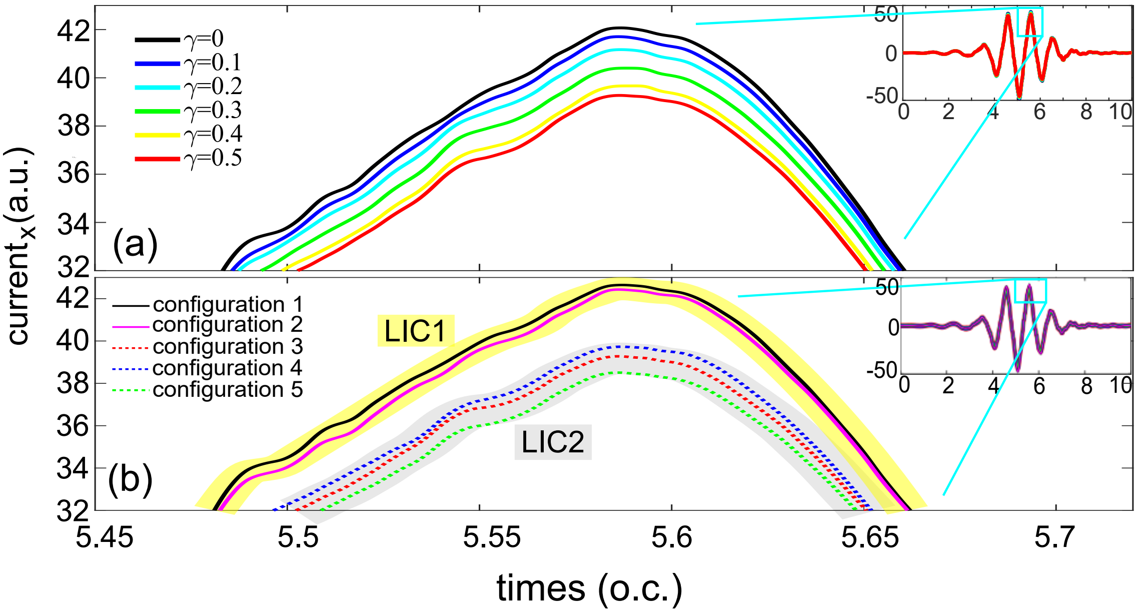

Supplementary Figure 15: **The variation of current amplitude with the hyperuniformity and the configuration change.** (a) Current amplitude comparison of different LICs with averaging over 10 configurations for each LIC. (b) Comparison of the laser-induced current amplitude between configurations 1∼5. The inset displays the overall appearance of currents, and the laser parameters are consistent with Fig. 2 in the main text. Source data are provided as a Source Data file.

Supplementary Notes

Supplementary Note 1: Energy spectra and eigenstates

By diagonalizing the Hamiltonian matrix in 2D discrete real space, we can obtain the eigenvalues and eigenstates of each configuration, and the occupied states will be the initial state of the time-dependent evolution. The energy spectra of configurations 1∼5 are shown in Supplementary Figs. 1(a)∼1(e) in turn, where the red dots correspond to the occupied valence band (VB), and the blue dots mark the unoccupied states in the conduction band (CB). Here, we perform the Hamiltonian diagonalization in a discrete circular space to avoid the interference of the CB by free electronic states in the boundary vacuum region, which has little effect on HHG. It can be observed that the gaps of the different configurations do not differ much, and the VB energy width of the one local isomorphism class (LIC) is the same. Comparing different LICs, the VB width of LIC2 exceeds the LIC1 (Penrose LIC) bandwidth (the gray area in Supplementary Figs. 1(a)∼1(e)), which is related to the electron localization caused by the vertex environment (VE) in LIC2.

In the 1D single-particle framework, the electronic states are all extended in periodic systems while localized in disordered systems [1]. Quasiperiodic systems lie between periodic and disordered systems, and there are particularly critical eigenstates for which the typical spatial distribution of wave functions decays to zero, then returns to larger values, and then decays alternately again. Ma *et al.* [1] used the rotational symmetry to simplify the tight-binding Hamiltonian of the Penrose-tiling center model and determined the coexistence of local, critical, and extended states by the localization analysis. To verify the rationality of our models, we analyze the electron localization behavior of configuration 1 in the Penrose LIC (LIC1), with the inverse participation ratio (IPR), the generalized first moment (GFM), and the generalized second moment (GSM) in real space.

For the ith eigenstate $\psi_i(x, y)$, the IPR I_i is,

$$I_i = \iint dxdy \, |\psi_i(x, y)|^4 \, . \tag{1}$$

IPR measures the number of atoms occupied by $\psi_i(x, y)$, and for the smaller value, the wave function is more extended; otherwise, the more localized it will be. Ma *et al.* [1] studied the tight-binding center model with N lattice points and concluded that the IPRs of the local, extended, and critical states are larger than 0.01, less than $1/N$, and between $1/N$ and 0.01, respectively. In our model with N_{atom} atoms, the conclusion that the local electronic states' IPR $I_i > 0.01$ still applies, but when IPR is less than $1/N_{atom}$, some states are not strictly full-space extended. The reason is that in the discrete real space, the electron wave packet is not only bound to the atomic position as in the tight-binding framework but has a certain width, which reduces the average distribution of the wave packet at each atom and makes the IPR of the extended state deviate to $1/N_{atom}$. By choosing IPRs that are small enough, we can similarly pick out the strict extended eigenstates.

The GFM M_i and GSM S_i of $\psi_i(x, y)$ are defined as,

$$M_i \equiv \iint dxdy \sqrt{x^2 + y^2} \, |\psi_i(x, y)|^2 \tag{2}$$

$$S_i \equiv \sqrt{\iint dxdy \, (x^2 + y^2) \, |\psi(x, y)|^2 - \left[\iint dxdy \sqrt{x^2 + y^2} |\psi(x, y)|^2\right]^2} \tag{3}$$

For an eigenstate, the GFM describes the average radius $< r >$ in the polar coordinate, and the GSM measures the extension in the radial distribution [1]. In addition to IPR, the GFM and GSM of different types of states also vary greatly. The GSM of the localized state is small, and it can be determined whether the state is localized at the edge or inside by the GFM value. The GSM of the extended state is large and covers the GFM. Many interesting distributions, such as ring wave functions, can be found for critical states according to the combination of the GFM and the GSM.

In Supplementary Figs. 1(f) and 1(g), we calculate the GFM and GSM for each eigenstate of configuration 1, respectively, represented on the IPR in the form of colors. For most unoccupied states on the CB, the IPR is small, and the GSM is large, corresponding to the extended states. For localized states, the IPR $I_i > 0.01$, and the GSM is small. Except for the 471st, 762nd, and 763rd eigenstates (localized at the center of the system, as in Supplementary Fig. 1(h) for the 471st), the GFM of the remaining localized states is large corresponding to the localization around the boundary, which is possibly from the broken lattice symmetry. The IPR between 0.01 and $1/N_{atom}$ belongs to a critical state, and its typical wave function distribution, e.g., the 249th eigenstate, is shown in Supplementary Fig. 1(i). For sufficiently small IPRs, the wave functions are extended with the entire spatial distribution, as shown in Supplementary Fig. 1(j). Therefore, the local, critical, and extended states coexist in our current model, which is consistent with the results of the tight-binding model [1] and proves the rationality of our model.

As mentioned above, the VB bandwidth of LIC2 is larger than that of LIC1, which we briefly discuss here. In Supplementary Fig. 2 (a), we compare the eigenvalue near the VB top of configuration 1 (black dots) and configuration 3 (red dots) and find that the energy of 938th∼975th eigenstates in configuration 3 exceeds the VB of configuration 1. When studying the light localization of generalized Penrose optical quasicrystals, Lin *et al.* [2] found that different LICs have characteristic vertex environments (VEs) (16 kinds in total), among which four special VEs, X, Y, Z, and ST, are closely related to the localization, as shown in Fig. 5a of the main text. In LIC1 (the Penrose LIC), the above four kind of the special VEs do not exist [2]. Therefore, in our model, the electrons in LIC1 are mainly located around the boundary, possibly related to the broken lattice symmetry. In LIC2 (taking configuration 3 as an example), there exists three kinds of the special VEs (Y, Z, ST), seeing Supplementary Figs. 2(b). By calculating the sum of electron densities of the 938th∼975th eigenstates, we can see that LIC2 eigenstates beyond the VB top of LIC1 are mainly localized in their three kinds of special VEs, as shown in Supplementary Fig. 2(c).

Supplementary Note 2: The electron diffraction spectroscopy

The X-ray and electron diffraction spectroscopy (EDS) of crystals show periodically arranged Bragg diffraction peaks, whose distribution depends on the crystal symmetry and has the symmetry (C_n, $n = 1, 2, 3, 4, 6$) allowed by crystallography. Diffraction in an amorphous form corresponds to a series of diffuse spots. Using the EDS, Shechtman [3] first observed the sharp Bragg peaks with the C_{10} symmetry of icosahedral phase in the quenched Al-Mn alloy, which opened the study of quasicrystals. Therefore, the EDS is essential for describing the quasiperiodic long-range order and symmetry.

Like 1D Fibonacci quasicrystal [4], we can obtain the 2D generalized Penrose quasicrystals' EDS $|F_2(k_x, k_y)|$ in k-space by the Fourier transform,

$$F_2(k_x, k_y) = \frac{1}{N_{atom}} \sum_{n=1}^{\mathrm{N}_{atom}} \exp\left[i(k_x x_n + k_y y_n)\right]. \tag{4}$$

The summation covers all atomic positions $\mathbf{r}_n = (x_n, y_n)$ in the 2D quasicrystal. It is worth noting that Eq. (4) effectively describes the EDS for our simple quasicrystal model, where only vertices are decorated with a single type of atom. For more complex structures with varied atom types and arrangements, additional corrections for atom types, positions, and disorder are required, as shown in Ref. [5]. By changing the parameter $\{\gamma_i\}$ of 5-grid, the vertices $\{\mathbf{t}\}$ in the cell plane can be obtained by the generalized dual method (GDM), and the atomic position $\mathbf{r}_n$ can be determined by enlarging the side length of the rhombus. Configurations 1 and 2 (configurations 3 and 4) belong to LIC 1 (2) and have the same EDS, as shown in Supplementary Figs. 3(a) and 3(b) (Supplementary Figs. 3(c) and 3(d)), respectively. For the different LICs, the overall difference is not obvious. We further enlarge each EDS in the range of the k_x, $k_y \in (-2, 2)$ a.u. as shown in Supplementary Figs. 3(e)∼3(h). For LIC1, a series of diffraction peaks lie on a line, constituting a series of rhombuses, as marked by the red dashed lines in Supplementary Figs. 3(e) and 3(f). However, in the EDSs of LIC2 (Supplementary Figs. 3(g) and 3(h) for configurations 3 and 4), the rhombus structure disappears. It is not easy to distinguish the EDSs of the above two LICs, which we expect can be achieved by the high-order harmonic generation (HHG).

Supplementary Note 3: The HHG for different intensities, wavelengths, polarization angles, and circular polarization

We pointed out in the main text that for the given star vector, the HHGs, driven by linear polarization, and their phases of different local isomorphic configurations are almost identical, as shown in Supplementary Figs. 4(a) and 4(b). In this part, we further confirm that this conclusion still holds for different laser intensities, wavelengths, and polarization angles.

First, we simulate the HHGs of various configurations at different laser intensities. When the laser intensity increases from $I_0 = 1.0 \times 10^{13}$ W cm^{-2} to $I_0 = 3.5 \times 10^{13}$ W cm^{-2}, the harmonic radiations of the configurations in one LIC are still almost identical, as shown in Supplementary Figs. 5(a) and 5(b) (corresponding to LIC1 and LIC2 respectively). Meanwhile, we find that the cutoff energy of the first HHG plateau is gradually becoming larger with the increasing laser intensity, as shown by the black arrows in Supplementary Figs. 5(a) and 5(b). In Supplementary Fig. 5(c), we compare the HHGs of different LICs, which have similar shapes and cutoff positions, but there are obvious differences in the yields for some photon energy. Especially when $I_0 > 2 \times 10^{13}$ W cm^{-2}, the harmonic yield of the 11th to 19th orders in configuration 1 is clearly higher than that in configuration 3. By further expanding the variation range of I_0, we find that the first plateau cutoff is proportional to $I_0^{1/2}$ (or E_0), similar to the crystal HHG [6].

In Supplementary Figs. 6(a) and 6(b), for diverse laser wavelengths, the HHG of different configurations in the same LIC also remains nearly identical. As shown in Supplementary Fig. 6(c), when the wavelength is short ($\lambda < 1.8$ μm), the difference between the two LICs' HHG exists but is not apparent. When the wavelength is

larger than 1.8 μm, the HHG yield in configuration 1 is higher than in configuration 3 in the 5 to 9 eV energy range. Therefore, the difference in the HHG yield can be used to distinguish different LICs, and this scheme has a wide window for different laser intensities and wavelengths. In addition, with the increase of laser wavelength, the cutoff energy of the HHG first plateau becomes smaller and the decrease tends to zero, as shown by the black arrows in Supplementary Fig. 6.

We also change the polarization direction of the linearly polarized laser by adjusting the angle θ between the polarization direction and the x-axis (as shown in Fig. 2b in the main text). The results still show that for any θ, the harmonic radiation of different configurations belonging to the same LIC tends to be identical (as shown in Supplementary Figs. 7(a) and 7(b)), while the difference in HHG yield of different LICs is modulated by θ (as shown in Supplementary Fig. 7(c)).

At the end of this part, we discuss the case of circularly polarized laser. Under circularly polarized laser, both molecular and crystal HHG have exhibited symmetry-related harmonic selection rules (HSR). In this scenario, small-sized configurations 1 and 2 (each comprising over 990 atoms) exhibit distinct $5k \pm 1$, and $k \pm 1$ ($k \in \mathbb{N}$) HSR, while the yields of the $10k \pm 1$ order harmonics are nearly identical (Supplementary Fig. 8(a)). Differences in HSR among configurations within one LIC are attributed to the finite size. Increasing the system's atomic number to 6171 (using configuration 1 as an example) aligns the harmonic selection rules with $10k \pm 1$, consistent with quasicrystal orientational order. As shown in Supplementary Fig. 8(b), the harmonic spectrum of LIC2 driven by circularly polarized laser also satisfies this conclusion, taking the enlarged configuration 4 as an example, whose HSR also tends to $10k \pm 1$.

Supplementary Note 4: The θ-dependent HHG for each configuration

As we have shown in Supplementary Fig. 7, for any θ, the harmonic spectra of two configurations in the same LIC are almost identical. Here, we complement the orientation-dependent HHG for configurations 1∼4. As shown in Supplementary Fig. 9, the θ-dependent HHG for different configurations in common LIC is almost identical. As highlighted in the main text, the finite size of small systems can cause minor fluctuations in harmonic intensity, which can be mitigated by expanding the system. As shown in Supplementary Fig. 10, we compare the harmonic yields of different orders in the plateau region (11th to 21st order harmonics) with various configurations to demonstrate that orientation-dependent HHG is similar in the same LIC but distinct in different LICs. The orientation-dependent HHG of all configurations is grouped into two clusters based on LICs, as highlighted by the light yellow (LIC1, configurations 1 and 2) and light gray (LIC2, configurations 3∼5) regions. Configurations within the same LIC show very similar HHG, while significant difference is observed between different LICs. This supports our conclusion that HHG orientation dependence can distinguish different LICs.

Supplementary Note 5: Intra- and inter-band harmonic components

HHGs in crystals are generally considered to arise from both intra-band currents and inter-band polarization. For the quasicrystal HHG discussed here, distinguishing these contributions in k-space is challenging due to the loss of periodicity. Fortunately, following the approach in Ref. [7], we can distinguish intra- and inter-band contributions in energy space by projecting the time-dependent wave function onto eigenstates. However, this approach is computationally intensive due to the extensive integration involved. To ensure computational feasibility, we have to reduce the system size. Here, we first tested the convergence of HHG in the current quasicrystal model for the number of atoms. For configurations 1 and 4, Supplementary Fig. 11 demonstrates the convergence of high harmonic spectra with the system's size under linearly polarized laser driving, reaching near convergence when the number of atoms $N = N_e = 456$. Therefore, in the following simulations of intraband and interband HHG, our model includes 456 atoms instead of the 991 atoms mentioned in the main text.

Supplementary Figures 12(a) and 12(b) show the intraband and interband HHG components for configurations 1 and 4. Comparing the relative intensities of intraband and interband HHGs shows that harmonics in the plateau region, with energies above MBG, arise from interband transitions, while lower-order harmonics involve contributions from both mechanisms. Comparing the inter+intra results with standard TDSE, we find differences in both low and high-frequency regions. This discrepancy arises from truncating higher-energy CB states for computational feasibility, which reduces both intraband and higher-order interband harmonic yields.

Thus, interband HHG dominates the differences in harmonic yields among LICs, influenced by VE-modulated eigenstate localization. Similar to crystals, we believe that in quasicrystals, interband harmonic corresponds to electron-atom scattering under an external field in real space. When the electron travels a sufficiently long distance, its coherence is affected by scattering from various VEs, leading to differences in harmonic yield across different LICs.

Supplementary Note 6: The effects of phason disorder on HHG

From Ref. [5], we know that phason flips can be easily performed for Q and D, as shown in Supplementary Fig. 13. Following the approach of Bugański *et al.* in Ref. [5], we start with a Penrose tiling (configuration 1, LIC1) and introduce phason disorder by randomly flipping Q and D. Supplementary Figures 14(a)∼14(c) show the

configuration with no flips, after 5 cycles of flips, and after 1000 cycles of flips, respectively. As the number of flip cycles increases, the rhombus pattern in the tiling becomes more random, in line with the literature [5].

In Supplementary Fig. 14(d), the phasonic disorder affects the harmonic yield in the plateau region. In configuration 1, after 5 flip cycles, the yields of the 15th, 17th, and 19th-order harmonics decrease and approach those of configuration 3 (LIC2). After 1000 flip cycles, the previously reduced harmonics are enhanced again, and their yields are comparable to those without phason flips. We also attribute these changes to the diversity and distribution of VEs. As shown in Supplementary Fig. 14(e), without flips, VEs are mainly D and J, with fewer others. Thus, scattering is dominated by D and J, resulting in less coherence loss and higher harmonic yield. After 5 cycles of flip, vertex environments become more evenly distributed among Q, M, K, L, and J, see Supplementary Fig. 14(f). The scattering of diverse VEs reduces electron coherence and lowers the harmonic yield. As iteration reaches 1000, in Supplementary Fig. 14(g), the predominant VEs are D and M, with fewer of the others. Consequently, electron coherence loss diminishes, and the harmonic yield increases. In addition, we can reasonably speculate that in the 2D cases, phason flips and disorder may change the orientation dependence of the HHG signal.

Supplementary Note 7: Effect of superuniformity on current amplitude

As the γ parameter increases, the amplitude of the current gradually decreases, which is consistent with the degree of superuniformity, as shown in Supplementary Fig. 15(a). It can be understood as follows: in the process of electron transport, electrons from different vertices will be scattered by a series of VEs. When the types of VEs are richer (or the superuniformity is weaker), the coherence between the currents of different electrons is weaker. Limited by the finite size of configurations in LIC2, the number of VEs will have fluctuations, it can also be reflected in the current amplitude changes. LIC2 has more types of VEs than LIC1, especially Y, Z, and ST [2], which leads to the localization behavior of eigenstates (Supplementary Fig. 2). These special VEs have more atoms with a compact arrangement, and the scattering effect on moving electrons is stronger, which makes the current amplitude weaker. According to the number of atoms, the ability to scatter electrons of the special VEs is ranked as ST $>$ Z $>$ Y. In LIC2, finite-sized configurations 3, 4, and 5 contain different numbers of each special VE, which is also reflected in the current amplitude, as shown in Supplementary Fig. 15(b). In Fig. 1, configuration 5 has the most 11 ST and 10 Y VEs, which hinder electron motion significantly and lead to the weakest current amplitude. Configurations 3 and 4 have 5 ST VEs, but the former also completely includes 5 Y and 3 Z VEs, so the current amplitude of configuration 3 is weaker than configuration 4.

Supplementary References

[1] Ma, P. & Liu, Y. Inflation rules, band structure, and localization of electronic states in a two-dimensional penrose lattice. *Phys. Rev. B* **39**, 9904–9911 (1989). URL https://link.aps.org/doi/10.1103/PhysRevB.39.9904.

[2] Lin, C., Steinhardt, P. J. & Torquato, S. Light localization in local isomorphism classes of quasicrystals. *Phys. Rev. Lett.* **120**, 247401 (2018). URL https://link.aps.org/doi/10.1103/PhysRevLett.120.247401.

[3] Shechtman, D., Blech, I., Gratias, D. & Cahn, J. W. Metallic phase with long-range orientational order and no translational symmetry. *Phys. Rev. Lett.* **53**, 1951–1953 (1984). URL https://link.aps.org/doi/10.1103/PhysRevLett.53.1951.

[4] Levine, D. & Steinhardt, P. J. Quasicrystals. i. definition and structure. *Phys. Rev. B* **34**, 596–616 (1986). URL https://link.aps.org/doi/10.1103/PhysRevB.34.596.

[5] Bugański, I., Strzałka, R. & Wolny, J. A new approach to phason disorder for a decagonal quasicrystal: the moment series expansion of the tiling distribution function for AlCuRh. *Journal of Applied Crystallography* **53**, 904–913 (2020). URL https://doi.org/10.1107/S1600576720006251.

[6] Ghimire, S. *et al.* Observation of high-order harmonic generation in a bulk crystal. *Nat. Phys.* **7**, 138 (2011). URL https://doi.org/10.1038/nphys1847.

[7] Guan, Z., Zhou, X.-X. & Bian, X.-B. High-order-harmonic generation from periodic potentials driven by few-cycle laser pulses. *Phys. Rev. A* **93**, 033852 (2016). URL https://link.aps.org/doi/10.1103/PhysRevA.93.033852.
